# Reliability and Agreement of Quantitative Pulmonary Imaging Biomarkers Between Ultra-Low-Dose and Low-Dose Chest CT: A Paired Intra-Individual Study

**DOI:** 10.3390/diagnostics16020327

**Published:** 2026-01-20

**Authors:** Da-Kyong Lee, Zepa Yang, Hwan-Seok Yong

**Affiliations:** 1 Department of Radiology, Korea University Guro Hospital, Korea University College of Medicine, Seoul 08308, Republic of Korea; naomi0135@naver.com; 2 Department of Computer Science, Soonchunhyang University, Asan 31538, Republic of Korea

**Keywords:** ultra-low-dose CT, quantitative lung imaging, airway and functional biomarkers

## Abstract

**Background/Objectives**: Ultra-low-dose computed tomography (ULD-CT) enables substantial radiation reduction compared with routine low-dose computed tomography (LD-CT), but its quantitative reliability across lung imaging biomarkers remains insufficiently characterized. This study aimed to assess the agreement and reliability of quantitative pulmonary imaging biomarkers between paired ULD-CT and LD-CT examinations. **Methods**: In this prospective study, 48 patients who underwent paired LD-CT and ULD-CT on the same day were analyzed. Whole-lung quantitative biomarkers were categorized into density-derived indices, airway structural metrics, and voxel-based functional biomarkers. Agreement between LD-CT and ULD-CT was evaluated using Bland–Altman analysis and Pearson correlation. **Results**: Density-based biomarkers demonstrated high concordance, strong correlations, and small systematic biases, indicating robust dose stability. Airway structural metrics showed clinically acceptable agreement with near-perfect reproducibility for cluster-based indices. Voxel-based functional biomarkers exhibited greater dose sensitivity but preserved consistent directional bias. Total lung volume showed excellent reproducibility with minimal bias. **Conclusions**: ULD-CT enables reliable quantitative lung imaging with clinically acceptable agreement across major biomarker domains, supporting its use as a dose-efficient platform for longitudinal and screening applications.

## 1. Introduction

Quantitative analysis of computed tomography (CT) has become an essential component for the phenotyping and longitudinal monitoring of chronic pulmonary diseases [1].

Guided by the “As Low As Reasonably Achievable” (ALARA) principle, chest CT protocols have progressively evolved from standard-dose CT to low-dose CT (LD-CT), which is now widely implemented in lung cancer screening programs [2]. Recent advances in detector efficiency and reconstruction technology have enabled further dose reductions, resulting in ultra-low-dose CT (ULD-CT) protocols that approach radiation exposure levels comparable to those of a two-view chest radiograph [3,4,5]. In particular, iterative reconstruction (IR) and deep learning-based image reconstruction (DLIR) techniques have substantially improved image quality by suppressing quantum noise while preserving structural detail [6].

In parallel with dose-reduction strategies, quantitative CT analysis has expanded to include radiomics-based feature extraction and deep learning-driven segmentation and prediction models, which have demonstrated utility in tumor grading, organ delineation, and outcome prediction across a range of clinical applications [7]. Although these approaches emphasize high-dimensional feature learning and predictive modeling, their performance fundamentally depends on the stability, reproducibility, and dose robustness of the underlying quantitative measurements.

Recent developments in photon-counting detector computed tomography (PCCT) have enabled further reductions in radiation dose while maintaining clinically acceptable image quality, which is of particular relevance for applications such as lung cancer screening. In this context, opportunistic assessment of additional imaging biomarkers has been explored, including coronary artery calcification (CAC), given the overlap between cardiovascular and lung cancer risk factors. A recent study reported that chest PCCT acquisitions at dose levels comparable to conventional chest radiography allow for visual and semiautomated CAC assessment. However, visual scoring tended to underestimate CAC severity at radiography-comparable dose levels, whereas semiautomated approaches appeared less affected by dose reduction. These findings illustrate that extreme dose reduction may differentially influence qualitative and quantitative imaging assessments, highlighting the need to evaluate the robustness and limitations of quantitative imaging biomarkers at radiography-comparable dose levels [8].

Taken together, these developments highlight that the clinical utility of radiomics- and AI-driven prediction models is critically dependent on the stability, reproducibility, and dose robustness of quantitative imaging biomarkers. Systematic noise-related bias or dose-dependent variability may propagate through feature extraction pipelines and adversely affect model generalizability, particularly in ultra-low-dose imaging settings. Consequently, rigorous validation of quantitative biomarker agreement across different dose levels represents a fundamental prerequisite for the reliable clinical application of radiomics and AI methodologies.

Despite these advances, prior studies have reported heterogeneous results regarding the quantitative equivalence between ULD-CT and LD-CT. While some investigations have described near-interchangeable measurements [9,10,11], others have demonstrated significant systematic bias, particularly in emphysema quantification and voxel-based air-trapping indices [12,13]. Many earlier analyses relied primarily on correlation coefficients, which quantify associations but do not assess agreement or identify systematic methodological differences [7]. Although the influence of CT noise on density-based metrics is well documented [14], relatively few studies have rigorously evaluated the magnitude and predictability of noise-induced bias across multiple biomarker domains using agreement-based statistical methods [15,16].

At ultra-low-dose levels, the intrinsic reduction in photon flux increases quantum mottle, leading to greater voxel-level variability in Hounsfield unit (HU) values [17]. This variability broadens the HU histogram of normal lung parenchyma and may spuriously shift voxels below the emphysema threshold (−950 HU), resulting in overestimation of the low-attenuation area percentage (LAA%) [18]. Noise-related effects are expected to be even more pronounced for voxel-based functional metrics, such as parametric response mapping (PRM) and emphysema–air trapping concordance (EAtC), which rely on voxel-wise comparisons between paired inspiratory and expiratory scans.

Accordingly, a comprehensive evaluation incorporating methods that assess both correlation and agreement is essential to determine whether ULD-CT can reliably substitute for LD-CT in quantitative structural and functional lung imaging [19]. In this context, the aim of the present study was to directly compare paired ULD-CT and LD-CT examinations acquired from the same individuals and to evaluate agreement and systematic bias in quantitative pulmonary imaging biomarkers using Bland–Altman analysis. Quantitative biomarkers were assessed across three domains—density-derived indices, airway structural metrics, and voxel-based functional markers, including air trapping, functional small airway disease (fSAD), and emphysema–air trapping concordance (EAtC)—to evaluate their reliability and potential clinical utility [20,21,22].

## 2. Materials and Methods

### 2.1. Study Design and Objectives

This prospective, paired, intra-individual study compared ULD-CT and LD-CT performed in the same imaging sessions. The primary objective was to evaluate the agreement between the protocols across multiple quantitative biomarker domains: density-based (LAA%, MLD, HAA%, subtracted mean HU, and LAAExp mean HU), airway structural (Pi10 and LAA cluster metrics ≥ 20 mm), and voxel-based functional biomarkers (AirTrap Volume, PRM-derived functional small airway disease [fSAD], and EAtC). Agreement was assessed using Bland–Altman analysis to characterize the systematic bias beyond simple correlation.

### 2.2. Study Population

This study was approved by the Institutional Review Board of Korea University Guro Hospital, and written informed consent was obtained from all participants. Fifty adult participants (aged ≥ 18 years) who underwent paired low-dose computed tomography (LD-CT) and ultra-low-dose computed tomography (ULD-CT) scans were prospectively enrolled. Two participants were subsequently excluded due to incomplete metadata or failed image reconstruction, resulting in a final cohort of 48 subjects included in the quantitative analysis.

The mean age of the included participants was 59.1 ± 12.9 years (range, 34–84 years), and the cohort consisted of 29 male and 19 female subjects. Patients with other major pulmonary diseases (e.g., interstitial lung disease, active infection, lung cancer, or postsurgical changes) were excluded to minimize potential confounding effects. All study procedures were conducted in accordance with the principles of the Declaration of Helsinki.

### 2.3. CT Image Acquisition

All CT examinations were performed using a single scanner (Aquilion ONE PRISM Edition, Canon Medical Systems, Otawara, Japan) during the same visit. Each participant underwent four scans: inspiratory and expiratory low-dose CT (LD-CT) and inspiratory and expiratory ultra-low-dose CT (ULD-CT).

Both protocols utilized identical scanning geometry, including 1.0 mm detector collimation and a lung-specific reconstruction kernel. The tube current–time product was reduced from 35 mAs for LD-CT to 17 mAs for ULD-CT, while the tube voltage was kept identical at 120 kVp across all acquisitions. All scans were acquired in helical mode using a predefined institutional chest protocol, resulting in minor inter-individual variation in spiral pitch (approximately 1.6–2.0).

For the ULD-CT protocol, a Silver-Beam (SB) filter was applied. The SB filter is a pre-patient energy filter designed to optimize the X-ray spectrum by selectively removing low-energy photons, thereby reducing radiation dose while preserving quantitative information.

All datasets were reconstructed using a vendor-supplied deep learning reconstruction (DLR) algorithm (Advanced intelligent Clear-IQ Engine; AiCE, Canon Medical Systems). DLR was selected for its documented capability to suppress quantum noise while preserving fine structural details, offering superior image quality compared with filtered back-projection or conventional iterative reconstruction techniques [23]. The selected DLR strength corresponded to the vendor-recommended clinical default for low-dose chest CT in routine practice and was applied uniformly across all acquisitions to minimize noise-related bias in density-based, airway structural, and voxel-level functional lung biomarker measurements.

Dose–length product (DLP) was recorded for each scan, and effective dose (ED) was calculated using a standard chest conversion coefficient of 0.014 mSv/mGy·cm. The mean DLP was 20.77 ± 2.35 mGy·cm for LD-CT and 10.93 ± 1.00 mGy·cm for ULD-CT, corresponding to effective doses of 0.291 ± 0.033 mSv and 0.153 ± 0.014 mSv, respectively.

The paired same-day acquisition design was intentionally adopted to minimize temporal physiological variability, such as changes in lung volume, airway tone, or transient inflammatory conditions, thereby isolating the effect of radiation dose reduction on quantitative measurements.

### 2.4. Quantitative Image Analysis

Quantitative image analysis was performed using a validated commercial software platform (Advanced intelligent Clear-IQ Engine; AiCE, implemented on the Aquilion ONE PRISM system, system version V10.12.SP0003E, Canon Medical Systems, Otawara, Japan), which applies a three-dimensional U-Net–based algorithm for automated segmentation of the lungs, lobes, and airways. Whole-lung quantitative biomarkers were extracted and categorized into three domains.

Density-based inspiratory biomarkers included total lung volume (TLV), low-attenuation area percentage (LAA%, ≤−950 HU), mean lung density (MLD), high-attenuation area percentage (HAA%), subtracted mean Hounsfield unit (HU), and expiratory LAA mean HU (LAAExp mean HU). Airway structural biomarkers assessed on inspiratory scans included Pi10 and LAA cluster metrics ≥ 20 mm, expressed as cluster count and percentage volume.

Voxel-based functional biomarkers were derived from paired inspiratory and expiratory scans and included air trapping volume (AirTrap Volume), parametric response map-derived functional small airway disease (PRM-fSAD), and emphysema–air trapping concordance (EAtC).

### 2.5. Statistical Analysis

All analyses were conducted in R (version 4.2.1; R Foundation for Statistical Computing, Vienna, Austria). Statistical significance was defined as a two-sided *p*-value < 0.05. Paired t-tests were used to compare LD-CT and ULD-CT biomarker means after confirming normality using the Shapiro–Wilk test. Pearson’s correlation coefficients were calculated to assess linear associations; however, agreement between LD-CT and ULD-CT was primarily evaluated using Bland–Altman analysis [19]. For each biomarker, the mean bias and 95% limits of agreement (LoA) were calculated to characterize systematic differences and measurement variability across dose levels. Rather than applying fixed clinically acceptable thresholds, agreement was interpreted in the context of same-day paired acquisition, relative LoA width, and the presence of systematic versus random bias, consistent with prior reproducibility-focused quantitative CT studies. This agreement analysis was applied to density-based, airway structural (Pi10 and LAA cluster metrics ≥ 20 mm), and voxel-based functional biomarkers (Air Trap Volume, PRM-derived functional small airway disease, and emphysema–air trapping concordance).

## 3. Results

ULD-CT demonstrated high quantitative agreement with LD-CT across density-based, airway structural, and voxel-based functional biomarker domains. Density-derived metrics showed the strongest robustness, airway structural measures demonstrated moderate but clinically acceptable reproducibility, and voxel-level functional indices exhibited greater variability but preserved monotonic LD–ULD bias profiles.

### 3.1. Density-Based Biomarkers

Density-based parameters, including LAA%, MLD, HAA%, subtracted mean HU, and LAAExp mean HU, showed strong LD–ULD concordance with small systematic attenuation shifts. These shifts were directionally consistent across subjects, reflecting predictable noise-related histogram broadening rather than segmentation failure. Overall, inspiratory lung density distributions were largely preserved at ULD-CT, supporting the clinical feasibility of ultra-low-dose CT for density-based lung assessment when combined with straightforward bias correction approaches.

**Figure 1:**
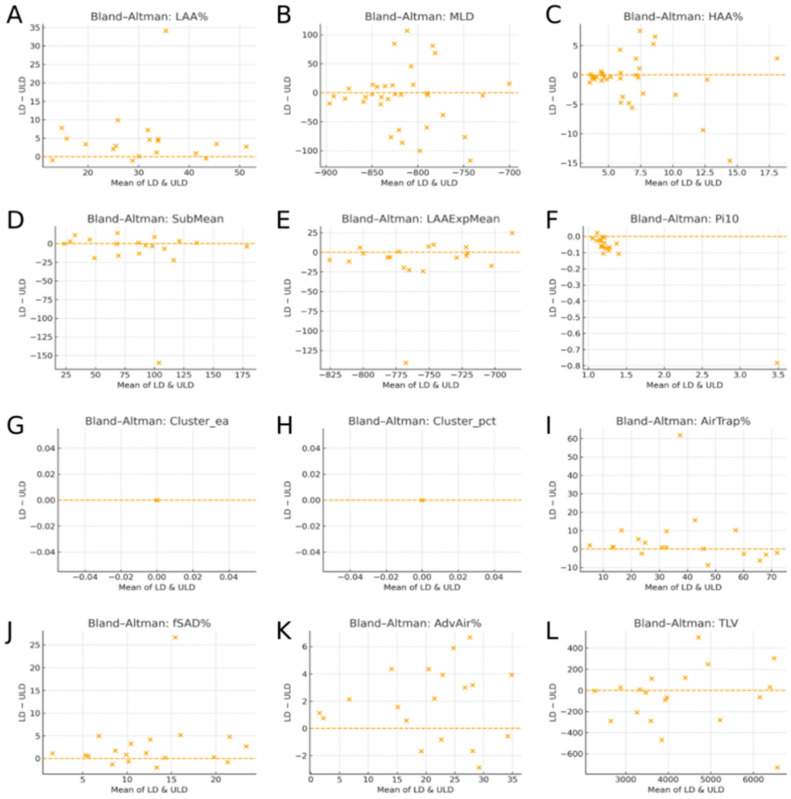
Bland–Altman plots comparing low-dose CT (LD-CT) and ultra-low-dose CT (ULD-CT) across twelve quantitative lung biomarkers. Density-based metrics are shown in panels (**A**–**C**), airway structural and cluster-based indices in panels (**D**–**I**), and voxel-based functional biomarkers and total lung volume in panels (**J**–**L**). The x-axis represents the mean of paired LD-CT and ULD-CT measurements, and the y-axis represents the paired difference (LD-CT minus ULD-CT). The horizontal dashed line indicates the mean bias, and the “X” symbol denotes the overall mean of the paired values. Across biomarkers, differences were symmetrically distributed around zero without proportional bias, indicating strong quantitative agreement between LD-CT and ULD-CT. In panels (**G**,**H**), data points are tightly clustered around the zero-difference line, indicating minimal systematic bias, in agreement with the very high correlation coefficients (r > 0.998).

The agreement between LD-CT and ULD-CT for all 12 quantitative biomarkers was further evaluated using the Bland–Altman analysis (Figure 1). Each Bland–Altman plot displays the average of paired LD and ULDCT measurements on the x axis, representing the central tendency of each biomarker, and the paired difference (LD–ULD) on the y-axis, indicating the magnitude and direction of the systematic bias. The narrow vertical distribution of differences around zero, without proportional trends, demonstrates high agreement across density-based, airway structural, voxel-level functional, and global volumetric biomarkers. The absence of proportional bias indicates that the magnitude of LD–ULD differences remains stable across the full measurement range, supporting a high level of agreement between the two dose protocols rather than value-dependent systematic distortion.

### 3.2. Airway Structure-Based Biomarkers

Airway structural metrics maintained moderate but clinically acceptable agreement across dose levels. Pi10 exhibited a small bias (−0.15 mm) and moderate correlation (r = 0.759, *p* < 0.001). LAA cluster metrics for regions ≥ 20 mm, expressed as count and percentage volume, showed near-perfect correlations (r > 0.998) and negligible bias, confirming the highly stable detection of larger structural abnormalities even at ultra-low doses.

Although airway wall estimation is more susceptible to noise and deep learning segmentation variability, all airway parameters demonstrated systematic and mono-tonic bias, implying that dose aware algorithmic refinement could further enhance their reproducibility and quantitative equivalence.

### 3.3. Voxel-Based Functional Biomarkers (AirTrap, fSAD, EAtC)

**Figure 2:**
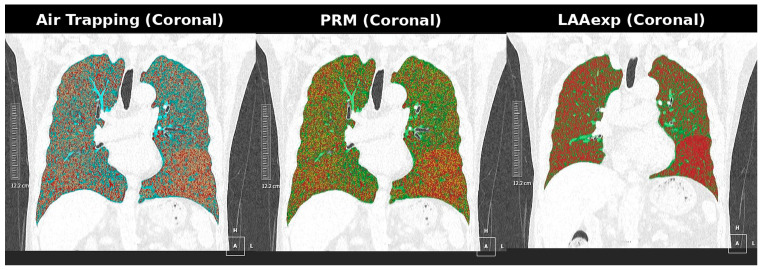
Case 1: Coronal AirTrap, PRM, and LAAExp maps.

**Figure 3:**
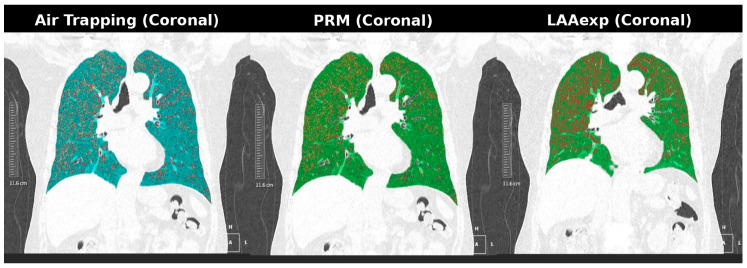
Case 2: Coronal AirTrap, PRM, and LAAExp maps.

Representative visual examples of voxel-based LD–ULD differences are shown in Figure 2 and Figure 3. These cases demonstrate that, although voxel-wise functional biomarkers exhibit greater numerical variability on ULD-CT, the spatial distribution of functional abnormalities remains preserved. This supports the interpretability of the ULD-derived functional maps within a harmonized quantification framework (Figure 2 and Figure 3).

Dose-robust example (Case 1). Paired coronal AirTrap, parametric response mapping (PRM), and emphysema extent (LAAExp) maps obtained from low-dose CT (LD-CT) and ultra-low-dose CT (ULD-CT). Lung parenchymal attenuation patterns, airway morphology, and emphysema distribution remain nearly identical between dose levels, illustrating high structural and density-based stability despite increased image noise at ultra-low dose.

Dose-sensitive example (Case 2). Paired coronal AirTrap, parametric response mapping (PRM), and emphysema extent (LAAExp) maps from LD-CT and ULD-CT demonstrate accentuated voxel-level variability on ULD-CT, particularly in AirTrap and PRM-fSAD maps. This example highlights increased noise-related HU dispersion in voxel-based functional biomarkers, while preserving consistent spatial patterns across dose levels.

Total lung volume (TLV) exhibited excellent reproducibility with negligible LD–ULD difference, confirming stable whole-lung segmentation under ULD.

A total of 50 paired LD-CT and ULD-CT examinations were analyzed using Whole-Lung-based quantitative biomarkers grouped into density-derived metrics, airway structural indices, and voxel-based functional measures (Table 1). Overall, ULD-CT demonstrated strong agreement with LD-CT, particularly for global and structural biomarkers, whereas voxel-level functional indices showed greater variability but maintained a stable and systematic bias.

## 4. Discussion

This study comprehensively assessed quantitative agreement between low-dose CT (LD-CT) and ultra-low-dose CT (ULD-CT) across density-based, airway structural, and voxel-derived functional biomarker domains. Despite inherently increased quantum noise at ultra-low-dose levels, most biomarkers demonstrated high reproducibility and predictable LD–ULD attenuation shifts, supporting the feasibility of ULD-CT for quantitative lung imaging in screening and longitudinal monitoring.

Density-based indices exhibited the strongest dose robustness, characterized by narrow limits of agreement and consistent attenuation shifts. These differences primarily reflected expected noise-related histogram broadening rather than anatomical or segmentation errors, indicating that density metrics can be rendered effectively dose-independent through straightforward linear correction or threshold adjustment. From a clinical perspective, such small and systematic shifts are unlikely to meaningfully alter emphysema categorization or longitudinal trend assessment.

Airway structural biomarkers demonstrated slightly greater variability, largely attributable to noise-amplified airway wall delineation and algorithm-dependent segmentation. Nevertheless, cluster-level metrics showed near-perfect reproducibility, suggesting that dose-aware airway modeling may enable reliable airway remodeling assessment even at ultra-low doses. Importantly, the observed LD–ULD differences were systematic and monotonic, indicating algorithmically predictable rather than random bias. This characteristic implies that, although absolute values may differ across dose levels, relative comparisons and longitudinal assessments remain suggestible to dose-aware refinement strategies such as noise-adaptive airway wall modeling, dose-conditioned calibration, or reconstruction-aware segmentation.

Voxel-based functional biomarkers showed the greatest sensitivity to dose-related noise; however, their LD–ULD differences remained directionally stable rather than erratic. Representative AirTrap, PRM-fSAD, and LAAExp maps (Figure 2 and Figure 3) demonstrate preserved spatial abnormality patterns despite voxel-level variability. Clinically, such behavior suggests that while absolute threshold-based interpretation may be affected at ultra-low-dose levels, regional disease distribution and longitudinal progression assessment may remain reliable after appropriate bias correction. This predictable bias profile highlights opportunities for noise-compensated reconstruction, adaptive thresholding, or harmonization algorithms to restore voxel-wise functional accuracy.

Collectively, these findings indicate a hierarchy of dose robustness, with density-based biomarkers demonstrating the greatest stability, followed by airway structural and voxel-derived functional measures. Across all biomarker domains, LD–ULD differences were systematic rather than random, supporting the potential for quantitative comparability rather than strict interchangeability without calibration. Accordingly, ULD-CT may serve as a dose-efficient platform for multi-domain quantitative lung imaging once appropriate harmonization strategies are applied.

In the broader context of quantitative CT imaging, recent advances have extended to radiomics-based feature extraction and deep learning-driven segmentation and prediction models. In parallel with algorithmic advances, recent state-of-the-art dose reduction strategies have combined spectral filtration and deep learning-based reconstruction to preserve quantitative accuracy at sub-millisievert dose levels. Notably, the integration of a Silver-Beam energy filter with deep learning reconstruction has been shown to substantially reduce radiation exposure while maintaining accurate cyst quantification and image quality in lymphangioleiomyomatosis and other cystic lung diseases [11]. These technical developments closely align with the acquisition and reconstruction framework adopted in the present study and further contextualize the feasibility of ultra-low-dose CT as a quantitative imaging platform when modern hardware and reconstruction techniques are appropriately applied. The performance of such approaches fundamentally depends on the stability of underlying quantitative measurements. In this regard, the present findings provide an essential methodological foundation by delineating the magnitude and structure of dose-related bias across multiple lung biomarker domains.

From a dosimetric perspective, the radiation dose achieved in this study falls well within established ultra-low-dose chest CT ranges. Using a standard chest conversion coefficient (effective dose = DLP × 0.014), the mean effective dose of the ULD-CT protocol was approximately 0.15 mSv, substantially below commonly accepted low-dose CT thresholds. According to guidance from the American Association of Physicists in Medicine, lung cancer screening CT is typically defined by an effective dose ≤ 1.0 mSv and a DLP ≤ 75 mGy·cm [4].

Consistent with prior reports, recent ultra-low-dose CT studies have demonstrated preserved quantitative performance at sub-millisievert dose levels. Golbus et al. reported a median effective dose of approximately 0.33 mSv (interquartile range, 0.33–0.35 mSv) using advanced reconstruction techniques [11]. Similarly, prospective randomized crossover trials have supported the feasibility of sub-millisievert chest CT in clinical populations [5], with earlier technical studies demonstrating diagnostic and quantitative utility at DLP values near 50 mGy·cm [23].

Several limitations merit consideration. Normal-dose CT was not used as an external reference standard; however, prior validation studies have consistently demonstrated strong concordance between LD-CT and conventional-dose CT, supporting the use of LD-CT as a surrogate reference to isolate the incremental effect of dose reduction. Additional limitations include residual noise-related variability in voxel-level functional metrics, use of a single commercial software platform, and a modest cohort size of 48 paired examinations, which limits generalizability, particularly for voxel-based analyses. Larger multi-institutional studies are warranted to validate dose-harmonized models across diverse scanners and patient populations.

Overall, these findings support ULD-CT as a promising quantitative platform across multiple lung biomarker domains, provided that dose-aware calibration and harmonization strategies are incorporated into clinical and research workflows.

## 5. Conclusions

Ultra-low-dose CT demonstrated strong quantitative agreement with low-dose CT across density-based and airway structural biomarkers, with small, systematic dose-related differences that are amenable to calibration or harmonization strategies. Although voxel-derived functional metrics showed greater sensitivity to increased image noise at ultra-low-dose levels, these measures retained consistent directional bias and preserved spatial abnormality patterns, supporting their potential utility for longitudinal and regional disease assessment after dose-aware correction. Collectively, these findings suggest that ultra-low-dose CT represents a clinically feasible and dose-efficient approach for multi-domain quantitative lung imaging, while further work focusing on standardized bias-correction frameworks, cross-vendor harmonization, and large-scale multicenter validation is required to establish robust clinical equivalence.

## Figures and Tables

**Table 1:** Comparison of whole-lung quantitative imaging biomarkers between low-dose CT (LD-CT) and ultra–low-dose CT (ULD-CT).

Category	Biomarker	LD-CT (Mean ± SD)	ULD-CT (Mean ± SD)	Bias (LD–ULD)	r	*p*-Value
Density-based	LAA% (−950 HU)	32.46 ± 13.78	27.80 ± 13.58	4.65	0.904	<0.001
	MLD (HU)	−845.3 ± 25.1	−855.7 ± 28.4	−10.4	0.96	<0.001
	HAA%	8.49 ± 3.62	9.07 ± 3.28	−0.58	0.915	<0.001
	Subtracted Mean (HU)	89.11 ± 45.50	94.37 ± 44.97	−5.26	0.779	<0.001
	LAAExp Mean (HU)	−761.23 ± 52.29	−751.77 ± 47.73	−9.46	0.882	<0.001
Airway Structure-based	Pi10 (mm)	3.43 ± 0.83	3.58 ± 0.96	−0.15	0.759	<0.001
	LAA cluster ≥ 20 mm, count	1.06 ± 6.94	0.72 ± 4.55	0.34	0.999	<0.001
	LAA cluster ≥ 20 mm, percentage volume (%)	0.11 ± 0.75	0.05 ± 0.32	0.06	0.998	<0.001
Voxel-based Functional	AirTrap Volume (%)	35.40 ± 21.38	31.56 ± 21.70	3.84	0.769	<0.001
	ParaMap_Volume (%)_WholeLung_fSAD	13.13 ± 8.33	10.72 ± 7.66	2.41	0.782	<0.001
	EAtC (%)—AdvAirTrap_Ovr_Emphysema (%)_WholeLung	22.81 ± 9.30	19.98 ± 9.45	2.83	0.961	<0.001
Global	TLV (cc)	4603.71 ± 1409.78	4590.18 ± 1361.85	13.53	0.978	<0.001

Density-based, airway structure–based, voxel-based functional, and global biomarkers are presented as mean ± standard deviation. Bias represents the mean difference between LD-CT and ULD-CT (LD–ULD). Agreement was assessed using intraclass correlation coefficients (ICC) and corresponding *p*-values.

## Data Availability

The data presented in this study are available upon reasonable request from the corresponding authors. The data are not publicly available because of institutional privacy regulations.

## Abbreviations

CT	computed tomography;
LD-CT	low-dose computed tomography;
ULD-CT	ultra-low-dose computed tomography;
DLP	dose–length product;
ED	effective dose;
DLIR	deep learning image reconstruction;
LAA	low-attenuation area;
MLD	mean lung density;
HAA	high-attenuation area;
PRM	parametric response mapping;
fSAD	functional small airways disease;
EAtC	emphysema–air-trapping concordance;
TLV	total lung volume.
